# Exploring Fragility: Industrial Delocalization, Occupational and Environmental Risks, and Non-Governmental Organizations

**DOI:** 10.3390/ijerph6030980

**Published:** 2009-03-05

**Authors:** Raquel Maria Rigotto

**Affiliations:** Federal University of Ceará, Brazil; E-Mail: raquelrigotto@gmail.com; Tel.: +55-85-3366-8045; Fax: +55-85-3366-8045

**Keywords:** Development, sustainability, occupational and environmental risks, non-governmental organizations, quality of life, public health

## Abstract

What is the role of non-governmental organizations – NGOs – in the process of industrial delocalization and socio-spatial redistribution of occupational and environmental risks? In an attempt to contribute to this debate, this study approaches the issue in a very specific socio-historical context, marked by recent accelerated industrialization in a small town in Northeast Brazil. Based on semi-structured interviews with leaders of four local NGOs, the way they perceive and value the risks introduced into the area and relations between industrialization and local development are analyzed. Findings show a strong adherence to the industrial plan by workers’ trade unions, whilst other NGOs are highly critical with regard thereto, but undertake no social or political activity regarding the issues they identify. This phenomenon is discussed in terms of the *modus operandi* of ideology and its strategies for symbolic construction, enabling a comprehensive reinterpretation of how capital also benefits, in its mobility, from local society’s fragility in organizing and protecting quality of life and public health.

## 1. Introduction

In advanced capitalism, one of the strategies adopted by corporations in order to guarantee accumulation has been to move their locations and organizations around the world. This process has been reflected in the socio-spatial distribution of occupational and environmental risks generated by production and consumption processes, with different implications for the health-sickness process of populations, classes and social segments [1–4].

There is a complex movement of de/re-localization of investments, in which process there are influences of an economic, political and cultural nature [5]:

- Expulsion forces, related to ecological reform under way in countries of origin, subject to stricter requirements in patterns of health, work and environment, in response to cultural and political transformations produced by society;
- Attraction forces, offered in new chosen territories, such as the price of land; ease of access and cost of water and power, amongst other natural resources; characteristics and cost of labor; tax exemption policies and infrastructural facilities offered by local governments;
- Welcoming forces for companies that relocate, built upon the condition of social privation of the communities in which they establish themselves and the hegemony of development ideology, shared by most of the local stakeholders, and which cause such corporations to be presented and greeted as the advent of “progress”;
- Protection forces, represented by the fragility of the executive branch and local civil society organizations in regulating and enforcing regulations governing work, health and the environment – which contributes, in practice, to preservation of the “paradise” in which new investment takes shelter;
- Reinforcement forces – those which, acting on a symbolic level, contribute to the covering-up of negative impacts of capitalist development and reproduce its ideology.

This study focuses on aspects of such protection and reinforcement forces in a specific socio-historical context, analyzing the participation of civil society organizations in the process of industrialization under way in a Northeast Brazilian town. There is resonance with authors such as Siqueira and Levenstein [6] who insist on the importance of interactions between the stakeholders of developed and developing countries in the definition of the risk profile to be introduced into the territory, as well as the magnitude of its effects or environmental control to be exerted over them. Studying the case of the Brazilian petrochemical industry, they show that workers, when organized, can even put pressure on the State to regulate and control environmental contamination.

Recognizing the mark of social and environmental *dumping*, this study also approaches the interpretation of Acselrad, upon stating that capital rewards, with its mobility, the least organized societies, with least capacity to resist the conditions it imposes and the negative impact generated thereby – and differentially distributed between the socio-environmental classes:

Capital therefore takes a considerable part of its contemporary strength from the capacity to delocalize, weakening less mobile social stakeholders – local governments and trade unions, for example – and undoing, through the blackmail of localization, urban or environmental government norms, as well as social advances. [7].

So how does this process unfold in the county of Maranguape – one of those which received the more than 600 new industries that came to the state of Ceará during the 1990s? Located in a semi-arid region of the Brazilian Northeast, where there is a concentration of time-honored regional inequalities, Maranguape has 88,135 inhabitants [8]. Superimposed upon an eminently agricultural and manual social and economic structure, and at the foot of a hill covered by a rare and special enclave of Atlantic Forest, eighteen new industries established themselves in its residential neighborhoods and downtown area as of 1995.

**Figure 1.**
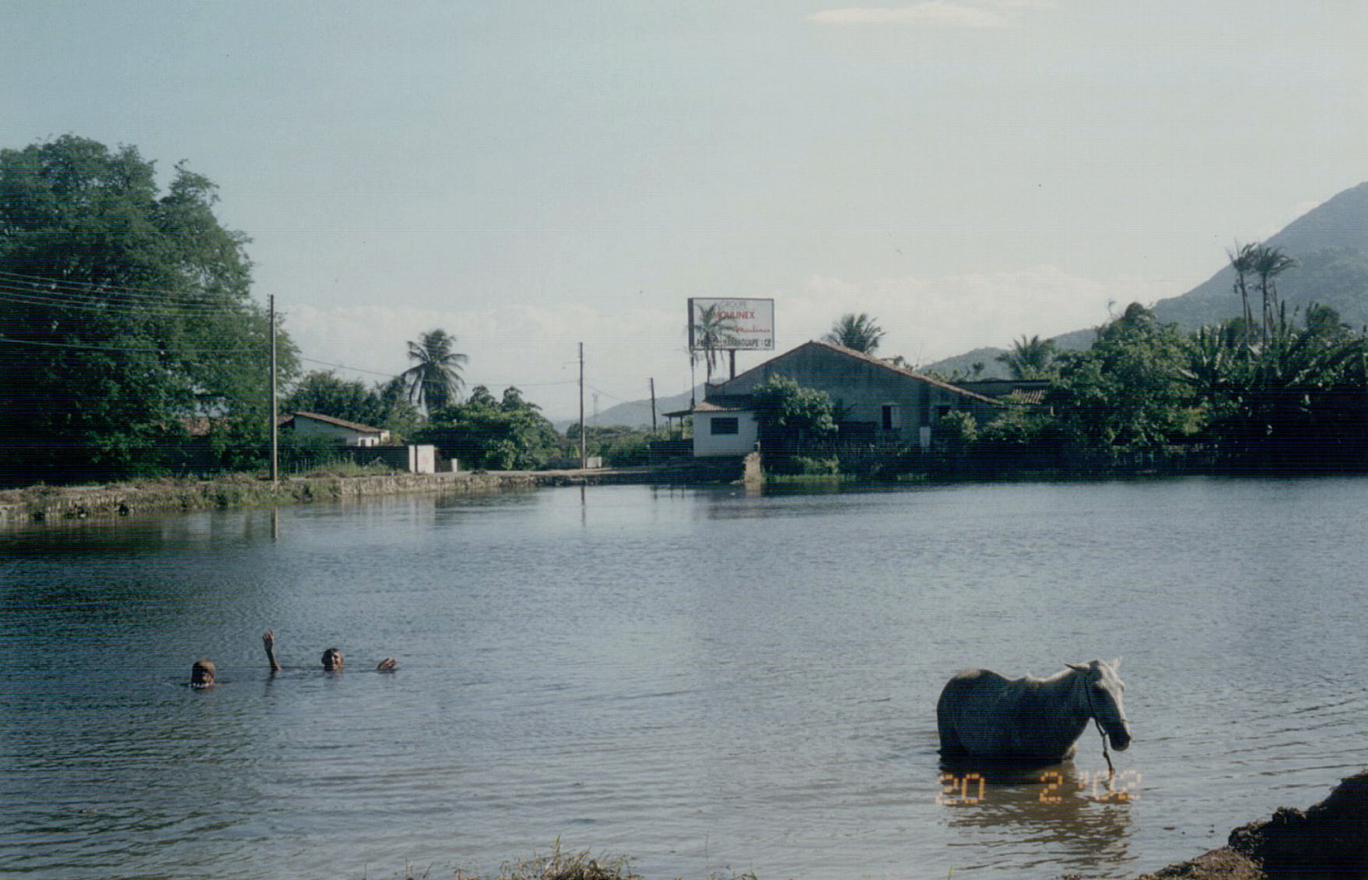
The Vavaú Reservoir, next to the electrical appliances factory, heavily used by the neighborhood community for several purposes. (Photo by the author)

**Figure 2.**
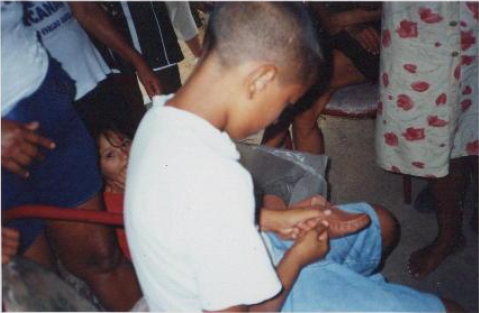
Child helping mother with domestic labor for footwear industry: fingers show the marks of stitching leather with a needle. (Photo provided by Iara Mar)

In a profound process of de/re-territorialization [9], these new structures generated a series of negative impacts – including the introduction of several important occupational and environmental risks. There are industries classified as having an average to high degree of occupational risk and potential for degradation of the environment. They produce metal cabinets, plastic packaging, chemical products, cardboard boxes, electrical household appliances, footwear, underwear and jeans. With the precarious nature of institutional regulatory mechanisms for the industry-health-environment relationship, such activities represent new occupational and environmental risks for the county, such as, for example [5]:

- The metal-mechanical industry, located opposite the Coach Station downtown, performs galvanization and painting of metal plates, and has used the residue from the liquid waste effluent plant to landfill the company’s main patio, causing pollution of soil and both surface and subterranean water.
- The chemical industry, originally from Rio Grande do Sul and which operates as a *joint-venture* with an Italian company, is located in a residential neighborhood, where it produces pigments and solvents for the footwear industry. This causes the frequent circulation of trucks with dangerous cargoes in residential areas, as the just-in-time paradigm is used. In turn, workers and local residents are exposed to inhalation of clouds of toluene and *n*-hexane, amongst other highly toxic solvents, causing drowsiness in children at school during emission peak times; in addition to the danger of fire and explosion – both of which happened in March 2005.
- The electrical appliances industry, with French and Brazilian capital, sends its liquid waste, including effluent from refrigeration tower, to a septic tank, without prior treatment. This is located in another residential neighborhood, on the banks of the Vavaú Reservoir, where there is a community laundry, while children bathe and boys fish (Figure 1)
- The footwear industry, also from Rio Grande do Sul originally, employs between 2,500 and 3,500 people, aged 22 on average, two thirds of whom are female. They work on their feet for eight or more hours per day. After being required by the Ministry of Labor to supply seats for employees, the company produced a technical report stating that it was better to work standing, and the case was sent to the appeal court. There is also considerable home-based work amongst women and children (Figure 2).

These are some elements of the context in which the study was performed. There follows a breakdown of the theoretical-methodological framework adopted, after which results shall be presented and discussed.

## 2. Results and Discussion

The first stage of the complete research project included the study of government industrialization policies and their history of implementation in Maranguape. The second stage featured a study of eight newly established industries in Maranguape with respect to working conditions and relations and the added occupational and environmental risks faced by public health policy makers. The third stage consisted of a survey of the understanding of different social segments – municipal authorities, workers, entrepreneurs and NGOs – regarding the process of industrialization and its relation to local development, including the perspective of public health and quality of life. The present article focuses on the perspective of the NGO segment.

To investigate how local collective subjects perceive and value these and other situations created by industrialization, an attempt was first of all made to determine the forms and instances of organization of civil society in Maranguape, by means of interviews with key informants. After a general panorama of this sector was outlined, it was possible to constitute a set of bodies that represent the diverse social segments in action in that socio-historical context:

- Clothing Industry Worker’s Trade Union, whose base is composed of workers from the footwear industry studied;
- UNECOM – Maranguape Community Bodies Union, a product of the strong local tradition in community movements, some of which arose from ecclesial communities in the 70s and 80s, today organized as neighborhood associations or cooperatives of small-scale farmers;
- Maranguape Citizen’s Council, which brings together eight county councils in which civil society is represented, as a form of participation in public administration and social control;
- Maranguape Community Social Economics Network.

The main leaders of these organizations were interviewed. These semi-structured interviews involved a script which enabled familiarization with the entity in hand (background, aims, composition, actions and challenges) and also to explore its view of the process of industrialization under way in the county, as well as its relationship with development. Interviews were recorded and the resulting text was analyzed according to the technique of discourse analysis [10–15].

The theoretical-methodological framework is, for the most part, based on the hermeneutics of depth, proposed by John Thompson [6]. This approach seeks to go beyond orthodox interpretation, by considering that the aim of investigation is a field pre-interpreted by the subjects that construct the field-object, and the researcher’s role is to re-interpret this field. Interacting with the symbolic conception of culture elaborated by Geertz, Thompson insists that emphasis on the symbolic nature of social life must be complemented by equal emphasis on the fact that symbolic forms are inserted in structured social contexts. He formulates what is called the “structural concept of culture”, which underlines both the symbolic nature of cultural phenomena and the fact that such phenomena are always a part of specific socio-historical processes and contexts – which involve power relations, forms of conflict and inequalities in terms of the distribution of resources. It is in these contexts and by means thereof that symbolic forms are produced, transmitted and received.

Thompson also develops a *critical concept of ideology*, which refers to “how the meaning serves, in certain circumstances, to establish and sustain power relations which are systematically asymmetrical – that I shall call domination relations. Ideology in the broadest sense, is meaning at the service of power” [6].

How may meaning establish and sustain domination relations? Thompson identifies some general modes of operation of the ideology, which may either overlay or reinforce one another, such as legitimization, dissimulation, unification, fragmentation and reification. These correspond with strategies of symbolic construction such as naturalization and universalization, assisting in reinterpretation of the discourse of subjects interviewed, and shall be commented upon later.

### 2.1. Clothing Industry Workers’ Union

The President of the Clothing Industry Workers’ Union, affiliated to the Trade Union Movement – as are the other trade unions to which workers in the town are linked, says: “I was invited to work at [the footwear factory] to organize the Trade Union for [the footwear factory] Group”. Invited? – I ask. By whom? He clarifies:

It was the company. People from the south are different from us. When they come, they want to form the trade union straight away, they want to get organized quickly, you see? Because if you’re organized into a trade union, everything’s easier to solve, through negotiation. So we founded the Maranguape trade union here, one in Russas and one in Iguatu. They have three factories here and one in the hinterland of Ceará state. So we took the chance to found unions in Pentecoste and Sobral. Today we have five new trade unions in the footwear industry here in Ceará.

The company wants to be organized, and workers notice: “there they have *their trade union, the factory’s. Anyone who works there has to pay!* I don’t know how you can have a trade union that you are forced to pay for!” Reveals Juciene, one of the footwear factory workers interviewed. (My italics)

The president speaks of the trade union as if it were his property: “I have another union”, “I joined the trade union movement”, “I passed it on to a friend”, “I put a son of mine on the job”, “I’m training a guy to take my place”. *I* and *the trade union* are practically synonymous. He decides, he gives guidance, he appoints and removes the president, he determined and prepares who will “inherit” the entity. When asked about the union’s activities, he lists a series of benefits – “when people get married, when a baby’s born, when someone dies...”

As there was no mention, amongst the union’s initiatives, of a common activity in such organizations – a campaign for a better salary – I asked about this:

Here in Maranguape, it’s hard to talk about better salaries. Most people don’t belong to a trade union; those who do get a minimum salary plus a little more. At the time of the convention, we bring everyone together and talk. It’s hard because for most of them it’s their first job, *it’s for them to learn.* Because the footwear industry is Ceará is relatively new, *people there had never seen a machine.* After they learn, the company has minimum salary levels *according to the capacity of each employee.*

(My italics)

The President accepts and reproduces the *modus operandi* of company ideology when he disqualifies the workers in order to justify the low salaries paid: “never seen a machine”, “it’s for them to learn”. He shows confidence in the fairness of the company’s remuneration policy, which establishes minimums “according to the capacity of each employee”. Yet during a later comment about the possibility of the company establishing itself in Maranguape, he contradicts himself: “production is the same [as factories in the South], quality is the same… They [the owners] are very happy: the workers are smart, they’re good!”

Even though he recognizes and confirms that salaries paid in the footwear factory in Maranguape are 30–40% lower than those paid in the South, he maintains the argument that workers are unskilled – “you have to know what you’re doing before you can demand anything”, adding to this the pressure of unemployment – “SINE has over 10,000 workers registered...”

I then asked about the relationship between the trade union and the members from the field, and he shows how he sees the dissatisfaction identified amongst the footwear factory workers interviewed:

We have direct contact with the *board of directors*: everything that goes on there we get to know. Every week I go there two or three times: there are problems involving mistreatment of workers by supervisors, *silly things* like that. First-time employees are always talking about rights, but they have obligations too. A company like this must have discipline! We have to inform workers about how the company words, so they’re not penalized on account of something silly. I go into the factory when I feel like it, I talk to whomever I wish, which helped reduce the number of issues being settled in court.

(My italics)

His contact, instead of being with the workers, is with “the board”. The “mistreatment” of workers – such as humiliation, which is so heavily charged for them in terms of suffering, as we have seen in their reports – is treated as “silly things”. He uses a business-like discourse, which underlines *obligations* to the detriment of *rights* – an illusion of a “first-time employee”, still virgin-like in terms of industrial experience. His discourse is full of examples of this inbuilt dominant ideology:

One *creature* turned up here, stayed 30 days without coming into work, because his son was sick: ‘The doctor said I had the right’! There’s no such right! What do you mean take us to court, boy? What are you going to complain about there? *I’m gonna have the firm fire you with just cause*, you go and negotiate, like lots do, bonuses and severance pay!

(My italics)

The workers: “a creature”. What rights do they think they have?! “A company like this has to have discipline!” The President of the Workers’ Union believes he is a part of the power exercised by the company – “I’m gonna have the firm fire you with just cause”, “I’ve already fired supervisors and managers there”. He refers to his relationship with the Superintendent as would a colleague and advisor: “I tell Mr Manoel [the Superintendent] to observe, he observes, then he comes along and says: `Chico, you’re right!’ ”. At other times, he takes on the role of a foreman: “When it’s a supervisor, the company asks Mr. Chico to go: ‘he has a supervisor’s position and has to be told off in front of you’” – he says, proudly.

I then examined his perception of the problems reported by workers interviewed at the footwear factory. He compares working without sitting down all day to his own experience in the textile industry, in which he “circulated”, and recognizes that working while standing in one place is “complicated”. Yet he says that “in a cashew nut plant in Pajuçara, they installed a seat and, after 60 days, employees were standing again, having abandoned the seats: ‘Mr. Chico, it gives us backache”. A solution for the problem of Repetitive Strain Injury – RSI is also “complicated”:

There’s also RSI. It’s tiring. We’ve had a lot of meetings with engineers to look at that situation. It’s complicated. We have to find the best method. If you change it and things get worse, then what? In 1955, I worked with two machines, when I left there were ten, a few days later there were fifteen: *modernization* is a nightmare. *Everyone* just cares about productivity. *All companies* are the same. It’s a *national* problem in Brazil.

He refers to modifications – and investments – that the company would have to make to solve the problem of prolonged orthostatism, and believes the results could be unsatisfactory, as with the cashew nut plant in Pajuçara. Problems suffered by workers are diluted by the reticence of being “complicated” and by *universalization* – one of the strategies for symbolic construction of the ideology described by Thompson – by modernization, by existing throughout the world, in all companies, in all of Brazil and, thus, they are *reified*. There is a clear feeling in the air that “that’s just the way it is” or, as the popular saying goes, “if there’s no remedy, the problem is already remedied”: in summary, the trade union does not see these issues as problems, fails to put them on the agenda and has no proposals for them.

Regarding exposure of workers to solvents contained in the glue used by the footwear industry, he, as with other workers interviewed, bases his arguments on incorrect information: “things are much better because the glue today is of a different type, it’s water-based”. And, once again, the risk generated in the context of the capital-work relationship is *generalized* to seem like everyday risk, *becoming banal* with *simplification* of solutions: “If you paint a door like this, you need solvent. You can’t make do without it, can you? Then you have to use a mask”.

When discussing the theme of industrialization and development, as one who participated in efforts to attract industry, he considers that they brought an important contribution, with generation of employment, and that there is no reason for them not to set up in Maranguape:

I was a representative here for 11 years, and I know how much we *sacrificed* to attract these companies, with collaboration from the State government and Raimundo Viana. Things *improved considerably* with the jobs we gained! Everything was in decline, all closed down. The [Footwear factory] has generated lots of jobs. I think it will be here for a long time, even with the end of fiscal incentives: where are you going to go? Productivity is the same, quality is the same... They’re very satisfied! People here are smart, good workers, I don’t know if that’s because they need the job… They’re more likely to close the Rio Grande do Sul factory than the one here.

In this way, the president of the trade union, a professional from the welfare and clientelism union organization, understands that new industries have benefited the city, bringing jobs for an unskilled population. He is compliant with the interests of the footwear company, reproducing their discourse and helping them to attenuate conflicts. Oftentimes, his identity is confused with that of the board of directors. On the other hand, he guarantees his own little empire – the trade union – where he exercises small powers to keep his “clients”. “We get by, without fighting, getting something out of it”. For whom?

### 2.2. Maranguape Community Organizations Union – UNECOM

UNECOM was founded in 1986, and its director today estimates that Maranguape boasts sixty associations of local inhabitants or small-scale farmers, of whom half are affiliated to UNECOM. When speaking of the relationship between industrialization and development, he first of all clarifies his concept of development: “For me, the basis for development is improvement in quality of life. Anything that compromises this quality of life doesn’t sound like development to me”. His arguments follow the same highly critical vein. Although he recognizes that new industry has alleviated the “serious problem of unemployment”, his evaluation is that the impact on the economy is greater on an individual plane, leaving few positive results for society:

What do we have in Maranguape? If the footwear industry closes, for example, what could happen? I’ll be brutally honest: I think it would be chaos. It’s a case of “bad with it, worse without it”, isn’t it? Now, I don’t know if that’s sufficient justification. From the economic standpoint, the impact is minimal. I believe the industrialization process in Maranguape, over the past ten years or so, has had a very small economic impact, because all that’s happened is generation of salaries, right? This salary enables people to buy things, feed themselves, get into debt – when you have a fixes salary each month, you take courage and buy something in installments. So you get into debt with a few stores and you eat a little better, at least you’re not concretely starving anymore. But the impact.... it doesn’t distribute income, there’s a very small impact on the county’s economy. If you add to all this the tax exemption policy… you end up with a situation in which what could have been added value for society, *disappears* in the form of tax exemption…

(My italics)

The Director of UNECOM also believes that quality of work is an essential component of quality of life – and, therefore, of development, in his opinion - choosing strong words to describe the capital-work relationships in Maranguape:

Not to mention that there are, as far as we know, very complicated relations. The capital-work relation has almost *feudal* characteristics, doesn’t it? It’s true! What we’ve seen is a complete *lack of respect* for human life, through *unlimited exploitation* of this unskilled labor force, which is a very cheap source of labor, with the *excuse* that they’re not qualified. But productivity indicators don’t confirm this lack of skills amongst the labor force.

(My italics)

Breaking with the hegemonic discourse, he is not convinced by the argument that local workers are unskilled, because there is evidence to the contrary – satisfaction with the performance of this labor force on the part of business owners - and sees the situation as unimpeded exploitation, disrespecting human life. Nor does he mince words when speaking of the relationship between industry and the environment: “a *total disrespect* for environmental issues, and a *complete absence* of social responsibility” He is also critical of the relationship between industries and the town community, characterized, for him, by failure to integrate:

You can’t see the mark of companies established in Maranguape, except for one or the other, especially in certain places – then we see that it makes a difference: the issue of integration with a place makes a difference! We don’t see company logos in a *single* social enterprise, nor in *any* facilities in the town. The companies are not even prepared to maintain a town square, which is a minimal cost and would have a very interesting effect on their marketing, wouldn’t it?

(My italics)

But he also perceives the low visibility of these problems:

In some cases, this *black box* has not yet *been opened* – it seems to me that it will be, little by little – but it’s still a black box. There are lots of things to consider, people’s *fear* of losing their job causes a lot of things to happen… But we have information, *comments* by workers, ex-workers from some companies, especially the [footwear factory], people who face problems, like pregnant women who have problems standing at their machines, but are not allowed to go for treatment, see? There are *reports* that I have heard personally, of people getting sick, going to the company doctor and not being allowed to go home, so they took the initiative of going to another doctor – the Public Health System (SUS) doctor, for example, and when they went back to the company with an SUS doctor’s note, the company doctor tore it up, discarded it, anyway…. So these things happen and *are not resolved*. Now, it’s complicated to discuss this, for example, because they’re very subjective reports…. If you call a person who makes a report like that to discuss it, they probably won’t, because people still lack *courage*…

(My italics)

The theme of the presence of industry in the town seems to be shrouded in a veil of silence, of “impending danger”, a taboo topic, nobody knows anything, they whisper about it. The examples of problems mentioned not just by this interviewee, but also by others, still seem only to scratch the surface of what I saw in these companies and heard from workers. Although interested, they’ve been unable to discover much to date. There is a veil that hides, silences and immobilizes. How is it possible to open this “black Box” if there is fear and insufficient courage? If all that is available are comments and personal reports? If the subject remains hidden in the passive voice in such sentences?

Nevertheless, later, the director indicates stakeholders who should be opening this Box – although he carefully chooses his words when referring to the municipal administration:

There is one complication in this story: those who should defend these issues, fight against such problems, are taking a very passive stance, which is the case of the trade union. I can see that the State has plenty of power – but specifically in this case, the Town Hall – this power is very limited. *There would have to be a mayor with considerable political will*, they’d have to be very clear-headed to *get into a fight like this*: it’s politically unfriendly to say that…. Now, we really have to do something urgently: we have to start discussing these relations!

(My italics)

The trade union is very *passive*, the mayor has not lived up to expectations. He notes here the perception of a power imbalance between stakeholders potentially in opposition in a “fight like this”, and underscores the urgency of, at least, commencing a debate: learning what is in the “black box”. However, still seeking more pondered expression, he clarifies that his project doesn’t exclude industry from the process of development in Maranguape, although this would require qualities very different from those identified today:

I’m not here suggesting.... defending that companies.... that Maranguape abandon the idea of industry. I think it’s part of the process, it’s something that’s here to stay and we can’t get away from it. Now, the relation *has to be* a relation that *has to be* as human as possible, as respectful of life as possible, right?

(My italics)

The project for development to which the director of UNECOM subscribes emphasizes the local – “the idea is to work with endogenous potential, with the internal potential of each place”. And several examples are given, such as the “bananas from the Lajedo Hills, that cut through the county, go to the fruit market (Ceasa), and then are bought by the population of Itapebussú, which neighbors the Lajedo Hills!”

I asked the director about the difficulty foreseen in advancing with the construction of local development project, and if companies intimidated the Town Hall:

They do intimidate, because the argument is very strong. No politician, and before being an administrator, the Mayor is, first and foremost, a politician, elected by popular vote – is willing to risk getting into a *fight*… So that’s it! A few months ago, perhaps just over a year ago, one of the companies, a lingerie manufacturer, threatened to leave the town. It was chaos! People demonstrated in the streets! So the opposition took advantage of the situation to take to the streets and announce the factory will close, and that Maranguape will have even higher unemployment, and that it’s the mayor’s fault. So the mayor found himself in a *tight spot*. There is one thing that interferes with all this: the foundation, so-called governability, is very fragile, because the government’s base is the Chamber of Representatives. There is no firm support, a society that can – for example, an organized community movement – that can, in a situation like this, say: “It’s OK, Mayor! Pick the fight because we’re here to support you!”

(My italics)

In the face of the threat of losing the company and losing political ground – the locational blackmail to which Acserald (2004) refers, the politician-mayor is *in a tight spot* and *retreats*, because there is insufficient support to *pick a fight* like this. In this way, for this community movement leader in Maranguape, although companies should not be excluded from the process, they are not the ones who will bring development – improvement in quality of life for the county: economic benefits occur for individuals who are employed, and are not distributed throughout society; labor relations have almost feudal characteristics, there is over-exploitation of supposedly unskilled labor; the environment is disrespected; cultural changes introduced lead to consumerism, violence and discarding of local potential – which, for him, form the foundation for development of the county. He perceives the urgent need for debate on this theme, to break the pact of silence and the veil of concealment established there, as well as the magnitude of capital of stakeholders who are on the other side of the social playing field. This is, in the words of Bourdieu, a heterodox and autonomous discourse, which is not linked even to the interests of the dominant, nor the subordinate, nor intermediaries.

But they do not operate in this field...

### 2.3. Maranguape Community Councils Chamber

The Community Councils Chamber, a successful experience that won awards from the Getúlio Vargas Foundation, hosts the Municipal Councils on Welfare, Education, Health, Sustainable Development, Employment, School Lunches, Guardianship of Minors and the Children’s and Adolescents’ Council, most of which have equal participation from the government and civil society. Its coordinator, Mr. Wilson, participates in the meetings of each of these councils, gaining a broad overview of the issues that have emerged in their agendas and of how these have been treated during meetings.

Mr. Wilson’s perception of the industrialization under way in Maranguape is that “there is a good side” and a “side that affects the community”. The good side is generation of income: the worker who, at the end of the month, will have his or her salary. The side that affects the community is that...

... the companies that come have only one commitment [to pay a salary]. We don’t see *any other* benefit. Several employees are cared for daily in the hospitals: they work with glue, with other toxins, in very hot buildings, so they faint… And then there are social issues: Not one company has a nursery school! *We’re aware of all this.* None of them contribute to any kind of social capital, social movements. *We see* that they generate income, but the workers are really being exploited. It’s exploitation! They don’t get a decent salary, they have no say in company matters. What’s more, that company doesn’t pay tax, because everyone knows why they come and set up in Ceará… and so… We still run the risk of, after fifteen years, everything closing down and moving to another county, don’t we?

(My italics)

The Coordinator of the Chamber of Councils *sees* both sides of the process of industrialization, and reveals a perception which is very similar to that of people linked to UNECOM, like the Mayor and the organization director interviewed. He recognizes the importance of generating income, but fills the other side of the scales with aspects that “affect the community”: low salaries, health risks at work, failure to comply with labor legislation, lack of support for social initiatives, careless treatment of the environment, lack of contribution to public funds and, above all, the permanent threat of moving away to another county. I asked him about the environmental impact of these new industries:

I *have no data* about that, I *don’t know*. It’s not yet *a concern for the county, nor for society at large, this issue hasn’t yet been addressed here* in the county. Although we know we might have companies here using chemical products, to make other products already manufactured here, but society isn’t concerned about that yet. *It’s not yet on the discussion table.* I think that’s why I don’t have any data on this.

(My italics)

Over the environmental issue, there falls the veil of concealment even upon this leader of a grass roots movement, capable of critically *perceiving* the process of industrialization in the town, but incapable of identifying risks introduced by new industries. Furthermore, he participates in the discussions that take place in all the organized councils in the county and tells us that this “has not been a concern for society” nor public administration – it’s “not yet on the discussion table”.

There is a similar picture with regard to the health problems caused by such work. I ask if, on the Health Council, problems like the one he had mentioned are discussed, that of people intoxicated by glue. He states that it was covered *once*, because the *hospital was overloaded*, but that he doesn’t know “what things are like today: *it must be more under wraps…* I don’t know if the industries solved the problem... I *don’t have any recent data on that*”. The black Box, of which the director of UNECOM speaks, caught a glimpse of an opening, but was soon *closed*, and the problem disappeared from the agenda...

The Coordinator emphasizes one element of the cycle of concealment – terror, revealing expressions that that floated in the air of the town and factories. Like Dejours [17] he perceives the feedback relationship between unemployment and the subjugation of those who are employed:

‘Don’t complain because otherwise the factory will close up and you’ll lose your salary’; ‘don’t complain because otherwise we’ll fire you, there are fifteen or twenty people out there waiting’… Although we have a few factories we didn’t have before, there is still a large group of workers outside trying to get a job, and this *terrifies* those who are inside! Because, ‘look, you can’t complain, you have your trade union here’ – the trade unions are formed on the inside… they don’t come near to helping at all (evasive)...

I wonder how these issues constitute problems in Maranguape, if the workers didn’t receive any information about this during their training; if the Health System is not prepared to make such diagnoses; if the trade union doesn’t recognize these rights; if “we don’t have any data” about environmental impact”...

Thus, the Coordinator of the Community Council Chamber of Maranguape, shares with other local leaders a development project which reinforces agricultural traditions and the region’s potential. It incorporates industries in the project, provided they are “decent”. His evaluation is that, to date, they have had the positive effect of income generation for some people, but they have generated many other problems – some of which are only now being understood.

### 2.4. Community Socio-Economic Network

The Community Socio-Economic Network is being formed in Greater Fortaleza, with the aim of “organizing the poor, especially unemployed laborers”, to produce and trade in their own products in a “captive market of *prosumers*”: producers and consumers, who would make collective purchases in markets and exchange programs run by neighborhood associations, using their own currency – in the case of Maranguape, the Guape. “The local market drives development, and community economy seeks to occupy that space, which rejects a series of the values of a traditional economy, working with awareness, solidarity, citizenship, with the issue of participatory democracy, with public policies of a social nature”.

Ednaldo describes the difficulties he sees in opening up these new prospects for development in the county:

There is an outdated culture, like, let’s say, that *old concept that “development equals industrialization”.* Here is no exception to that rule. I think that, here in Ceará, the phenomenon of industrialization contributed to that considerably. And this culture has a *very strong impact on people’s awareness!*

(My italics)

The old (?) concept that “development equals industrialization) is updated and reinforced in local culture, by the industrialization stimulated by the state government, and this distracts people’s attention from the traditional forms of development towards industrial employment. I therefore asked Ednaldo to talk about his vision of the industrialization process in Maranguape and he began by recognizing the positive, yet limited role, of industry: in a context of social suffering, industries minimize the problem, at least for a *sector* of the population, offering a *minimum* salary. He then refers to the health problems faced by workers, relating them to intense exploitation:

The second thing is the impact on workers’ health. There are many complaints. There is a high degree of exploitation of these workers. That is very clear. Then there is the impact on their families. This is problematic: the issue of intensification of exploitation, which is a pattern of behavior, let’s say, *on the part of industry in general*. I think these company’s profit margins are phenomenal!

As with the other critical stakeholders interviews, he also unabashedly criticizes the lack of commitment from these new industries to the town and its population: “these companies have *no relationship* with the community; they contribute *absolutely nothing”*. He shows his indignation with fiscal incentives and encouragement granted by those in power to new industries – and the inequality in treatment of different social groups:

The government’s investment in order for these companies to stay here, the concession, is enormous, and I consider it an anti-democratic stance, because there are no concessions to small-scale industries! The forecast is that, when the tax concession period runs out, they will move on to another development paradise... desenvolvimento...

The path ahead for this paradigm shift, in his view, is the organization of society:

The democratic process depends largely upon the strengthening of civil society. Experience in Brazil, in Latin America, has demonstrated that there are considerable changes towards increasing the people’s quality of life, in order to improve the services provided by the State, and even the governmental reforms so eagerly anticipated – Brazil and Latin America are still highly authoritarian – this will only be possible if there is a grass roots movement in society.

But this form of organization has not been the agenda for industrial-health-environmental relations in activities in Maranguape.

## 3. Conclusions: NGOs and the Risks Generated by Industrialization in Maranguape

**Figure 3.**
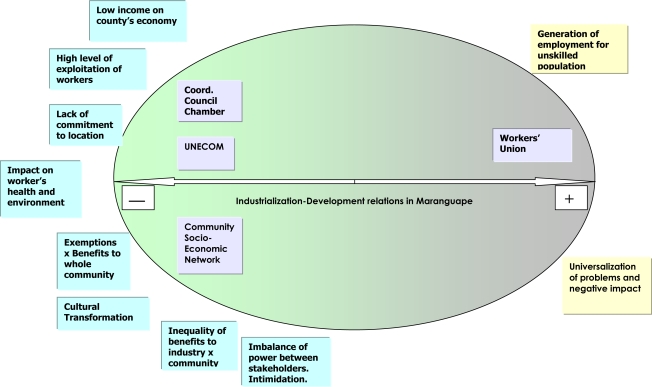
Positions of discourse of social stakeholders in Maranguape community regarding industrialization-development relations and symbols which sustain them.

Annex seeks to graphically represent the positions of social subjects interviewed with regard to industrial relations and development, showing how the perception of occupational and environmental risks generated by the new industries participates in such representations (Figure 3).

It is found that, on the one hand, there is the Clothing Industry Workers’ Union – clearly constituted by initiative of the footwear industry, which considers relations to be positive, inasmuch as companies bring employment for a population they consider “unskilled”. The generation of employment is the fair and just cause of support, founded on the rationale or sacred nature of work, which *legitimizes* industrialization ideologically. In the face of this “legitimate cause”, operation risks generated by the production process and repeatedly mentioned by workers, undergo, in the symbolic construction of the president of the trade union, a clear process of *naturalization* - another operational strategy of the ideology, in which situations that result from historic or social creations are treated as natural events. The technique of *universalization* is also used, showing situations or problems linked to the interests of a group as if they belonged to all, and *disguising* the domination relationships which permeate exposure to risk at work. In this way, in the hierarchy of symbolic values which the union president subscribes to, establishes or reproduces, these risks, like the workers’ complaints, are secondary in relation to the ‘benefits’ brought by the industries’ presence: he shares the ideology of development. These are the representations that guide the practices of the trade union, which hides and minimizes the risks and disqualifies the workers’ demands in regard thereto.

Thompson [6], upon questioning the understanding of the ideology as “social cement”, offers several elements which help one comprehend the implications of this stance by the trade union. For this author, the reproduction of the social order does not require a deep underlying consensus upon values and beliefs, provided there be sufficient *dissent* to prevent formation of an effective opposition movement. The president of the Maranguape Clothing Industry Workers’ Union – a professional body, upon so clearly consorting with the dominant ideology as a consensus, produces and disseminates dissent. Workers fail to identify, amongst the speeches and information to which they have access, anything which corresponds to their anxieties and dissatisfactions, and which is congruent with their new experience of the working world. There is no symbolic core of conformity in which they can elaborate their ideas and feelings, not even at the union. A further strategy may be identified here – that of symbolic construction and operation of the ideology – fragmentation, in which individuals and groups that may be capable of transforming into a real challenge to the dominant groups, are segmented and isolated. They therefore become unsupported. By confusing in his identity the representation of workers and the company’s ideology, the union president confuses the process of generation of symbolic forms of criticism and makes it more difficult for these to be organized in to an opposition movement.

The other three social organizations interviewed showed a very critical view of the role of industries in local development. They indeed recognize the benefit of income generation, but emphasize that its positive impact is limited: occurring only on the individual plane, and for a specific sector of the population. They point out the several negative aspects of new industries, such as the level of exploitation of workers – expressed in low salaries, failure to comply with labor legislation, lack of respect and poor working conditions. They criticize tax exemptions and the incentives offered to companies, both because the same is not offered to small local entrepreneurs, and because this makes the benefits they could potentially bring to the whole community, in the form of government income from taxes, disappear. They denounce the permanent fear that industries will move away. They identify cultural changes introduced by the new industries, related to consumerism, violence and drugs, as well as in family relationships. Overall, they conclude that these companies have benefited more than their town.

The occupational and environmental risks generated by these industries are felt by those bodies and are considered in the analysis: they all refer to the impact on health of workers and the environment as highly critical manifestations of companies’ ethics with regard to life, as an expression of their lack of commitment to the local community, and as the antithesis of the very development they were supposed to bring, as they compromise fundamental aspects of quality of life.

These organizations share another local development project, constructed over twenty years in the context of social movements, whereby industries could have a role to play, but not a central one, and they would be “decent” industries, as they put it. Thus, they do not recognize the “cause” of industrialization as being legitimate.

Nevertheless, none of them take the issue of industrialization and the problems derived therefrom as the object of action today. Throughout Maranguape, there is not a single NGO that has placed this problem on its agenda for mobilization and struggle.

Why is that? An initial element for analysis is provided by Tábara [18] and Freitas [19], when they remind us that the environment “itself” is not “problematic”. Changes therein only become “problems” when they are considered as such by society, when they affect society or are perceived thereby, when individuals or social groups define them as problems. As with all human anxieties or needs, environmental problems also require social construction or affirmation, which is subject to its own historic evolution in each culture, until they come into existence and generate needs, as explained by the Coordinator of the Community Council Chamber.

In order for environmental problems to be socially endorsed, they must be constructed and disseminated in the social sphere by several social stakeholders, until they are recognized by a broad range of individuals and institutions. It is then necessary to consider the incipience of the phenomenon of industrialization in Maranguape, and the social time necessary for different transformations produced by the installation of eighteen new factories in the city to be perceived and symbolically attributed value, until they are included in the social agenda.

One must also take into consideration that this perception varies between groups within society. According to the anthropological approach to perception of risks, the selection of a set of phenomena that constitutes danger, as well as perception of acceptable levels of risk, are social constructs, which vary between different cultures and in different social groups within each culture. Each individual or society filters which risks it wishes – or can! – fear, so as to lend support or coherence to its own form of perception and its own values (Douglas and Wildavsky, apud Freitas [3]). In this way, in symbolic production in Maranguape, factors such as quality of life, respect for health and the environment compete with the value of a job and the meaning of inclusion in society, even though such integration may be “fictitious”, as Bourdieu [20] puts it.

Availability, quality and access to information are also part of this process of formation of social experience, in which risks are given values, as proposed by a sociological approach: how can the citizens of Maranguape become aware of the risks generated by factories, if local public authorities fail to monitor air or water quality; they neither investigate nor release information about health problems related to work or environmental contamination, and if there is a lack of transparency regarding inspection and control procedures? As proposed by Mol [21], the generation of sufficient and reliable public environmental is a basic task of the State for support of open and democratic political processes. The omission of such data, on the other hand, feeds the process of concealment of damage to the development process and delays the organized response of society – a delay which favors the investors who, in the fast and transitory pace enabled by Globalization, advance towards generation of profit and risks, whilst benefiting from ten or fifteen years of tax benefits.

It is therefore possible to comprehend the “black box” mentioned by the director of UNECOM: state policies of omission and disinformation; business strategies for legitimization, naturalization and fragmentation form a pact of silence and a veil of concealment of the negative impact and damage of risks generated by the industrialization process. Even though community organizations committed to sustainable local development have a critical view of this process, they shall have to compete with the “infernal alternative” broadcast to the population: poverty or unemployment in industry, and conditions imposed thereby, submitting to what Acselrad calls “locational blackmail” [15]. This is where the capital of each collective subject – economic, symbolic, political, come into play – in the social sphere, as proposed by Bourdieu [2], and these forms of capital are profoundly unevenly distributed in Maranguape. The “terrified” workers are afraid of mentioning problems that inhabit work processes, and are unable to count on the trade union to be their voice. On Councils and in municipal administration, these issues “are not on the agenda”. Community organization that are critical of the process seek access to reports, as the authorities fail to generate or them or release data on environmental impact, health hazards etc.

If this is the case, we face an uneven playing field. And, as Thompson states, “when established power relations are systematically asymmetric, then the situation may be described as being one of domination” [6]. In the case of Maranguape, the economic, social, political and cultural context in which such domination is exerted brings specific aggravations, which contribute to the reproduction of development ideology. Capital benefits from it – especially from the fragility of civil society bodies in advancing towards implementation of projects. It is known that if and when the context becomes less favorable, industries may threaten and effectively withdraw from the location, moving to another territory in which these and other “locational advantages” are reproduced. This is a considerable challenge to Productive Reengineering under Globalization.
